# Management of implant plate exposure of silicone Ahmed glaucoma valve: a review of six cases

**DOI:** 10.3205/oc000047

**Published:** 2016-09-02

**Authors:** Avik Kumar Roy, Sirisha Senthil

**Affiliations:** 1 VST Glaucoma Center, LV Prasad Eye Institute, Kallam Anji Reddy campus, Hyderabad, India

## Abstract

**Objective:** To describe the management options for exposed silicone Ahmed glaucoma valve (AGV) implant.

**Methods:** This was carried out as a retrospective chart review at a tertiary care eye hospital in Southern India. Medical records of six subjects managed for AGV exposure from 2006 to 2013 were reviewed.

**Results:** All six eyes had explantation of the AGV and 3 of them had reimplantation in a different quadrant at a later date and the other 3 eyes were managed medically. All eyes had well controlled IOP at the last follow-up. The possible predisposing factors for exposure were improper conjunctival coverage, higher number of pre shunt surgeries and diabetes mellitus. Reimplantation was a challenge with scarred conjunctiva and the techniques used were conjunctival advancement, conjunctival relaxing incisions and contralateral conjunctival autograft. None had re-exposure but one eye had conjunctival erosion close to the limbus and was managed with scleral patch graft and conjunctival advancement.

**Conclusions:** Implant exposure is a serious vision threatening complication following glaucoma drainage device implantation. Explantation and timely repair can save these eyes from serious sequel. Reimplantation is a good option, however warrants close follow-up for complications like erosion or re-exposure.

## Introduction

Glaucoma drainage devices (GDD) have been increasingly used in the management of refractory glaucomas both in children and adults with good success rates. In approximately 2–7% of cases, part of the device is exposed outside the conjunctiva [1] with almost equal incidence of exposure rate in both the early (<6 months) and late (>6 months) postoperative period [2]. Causes of implant exposure and/or tube erosion are diverse, so is their management. Currently, we have very little understanding regarding this rare complication. Hence, the aim of our study is to discuss the options in the management of this serious complication.

## Case description

After receiving approval from the institutional review board, electronic and paper records were obtained for all patients who underwent Ahmed glaucoma valve (AGV) explantation at our tertiary referral eye care center between January 2006 and August 2013. All the explanted AGVs were silicone implants. A single surgeon (SS) managed these complications in this series. Medical records were reviewed with regard to demographics, preoperative diagnoses, co-morbid conditions, intraoperative details, postoperative complications, management strategies and outcomes. Table 1 (Tab. 1) summarizes the demographic details, ocular history, and outcomes of the six eyes with implant exposure. Figure 1 (Fig. 1), Figure 2 (Fig. 2) and Figure 3 (Fig. 3) show preoperative, intraoperative and postoperative photographs of the study eyes. 

In the 1^st^ case, the patient was referred to us with a totally extruded implant (Figure 1a (Fig. 1)) at the limbus without any patch graft or anchoring suture in her only seeing eye. The extruded implant was explanted and the fistulous opening to anterior chamber was closed with corneal patch graft and interrupted 10-0 nylon sutures. Reimplantation was not possible in the same sitting because of severe hypotony. Severe conjunctival scarring in the anterior conjunctiva 5–6 mm from the limbus was noted in all the quadrants due to previous multiple vitreo-retinal surgeries making the choice of reimplantation difficult. Considering the unpredictability as well as the risk of phthisis in an eye with previous retinal detachment, transscleral cyclophotocoagulation was not a choice in this patient. In view of medically uncontrolled IOP, reimplantation of a smaller pediatric sized Ahmed glaucoma valve (FP8 model) was performed 2 months later in the inferotemporal quadrant with a large donor scleral patch graft (Figure 1b (Fig. 1)) and large (12x15 mm) conjunctival autograft from the non-seeing contralateral eye. The conjunctival autograft was secured with fibrin glue (Tissel, Baxter, Westlake Village, Calif.) over the scleral patch graft and the edges were sutured (Figure 1c (Fig. 1)) to the host conjunctiva with multiple interrupted non-absorbable sutures to ensure stability, vascularity and graft survival. Postoperatively she was started on oral doxycycline (100 mg twice daily for 6 weeks) to hasten conjunctival healing and prevent collegenolysis and conjunctival necrosis [3]. She presented 9 months later with 0.5 mm anterior conjunctival erosion and tube exposure (Figure 1d (Fig. 1)) for which she underwent scleral patch graft with conjunctival advancement. She is doing well at 3 years post surgery with stable implant (Figure 1e (Fig. 1)), well-controlled IOP and stable vision and no exposure after that episode. Ideally, during an implant surgery, using a free conjunctival autograft over a donor scleral patch graft is not recommended owing to the possibility of compromised perfusion and graft survival. However, when adequate care and precautions are taken, this technique could help manage a difficult situation as encountered in our patient. 

The 2^nd^ case was an 11-year-old child with steroid induced glaucoma with an inferotemporal FP7 implant operated elsewhere, that extruded two months after surgery (Figure 2a (Fig. 2)). During explantation, we noted that the implant was improperly anchored, was displaced over the lateral rectus muscle and the patch graft was absent. Reimplantation with smaller FP8 implant (Figure 2b (Fig. 2)) was performed one month later in the superotemporal quadrant. The eye experienced a hypertensive phase in the 3^rd^ postoperative week; the IOP was controlled with one antiglaucoma medication till the last follow-up at 8 years and there was no recurrence of erosion or exposure of implant. 

The 3^rd^ and 4^th^ case represent the left and right eye of a 47-year-old lady with uncontrolled uveitic glaucoma and severe scleral thinning. She had undergone primary AGV implantation in superotemporal quadrants in her right and left eye two weeks apart. She presented with exposure of AGV implant 6 months later and was referred to us. Absent conjunctival coverage over half the implant plate with thinning of underlying sclera was noted. The IOP was 10 and 12 mm Hg in the right and left eye respectively. She underwent explantation of AGV with sclera patch graft and conjunctival advancement. The IOP was medically controlled in both the eyes after explantation until the last follow-up at 7 years. 

The 5^th^ case had glaucoma drainage implantation for post-penetrating keratoplasty glaucoma, presented with hand movement vision and digitally low IOP following trauma, which had displaced the implant and partly extruded with corneal decompensation. This was managed by explantation of the implant and the IOP was under control with one antiglaucoma medication till 1^st^ week after which he did not come back for a follow-up with us, however underwent corneal transplant elsewhere.

The 6^th^ case underwent combined cataract and AGV implantation in his only seeing right eye, following which he experienced five episodes of implant exposure, managed by various combinations of patch grafts including amniotic membrane, conjunctival autograft, and buccal mucosal graft at multiple centers. At presentation to us, the body of the AGV was totally extruded (Figure 3a (Fig. 3)) along with the buccal mucosal flap overlying it with anterior chamber exudates, resolving vitreous hemorrhage and hypotony. Suspecting an endophthalmitis, he underwent emergency explantation of extruded implant and closure of fistula together with intravitreal antibiotics, the culture and smear were negative for any organism and the inflammation resolved with appropriate medical treatment. He underwent reimplantation of AGV in the inferotemporal quadrant 1 week later. Postoperatively, the implant was stable (Figure 3b (Fig. 3)) with the IOP under control with one antiglaucoma medication till the final follow-up. There was no exposure noted after reimplantation in that eye.

## Discussion

Among the various complications related to AGV implantation, plate exposure and tube erosion can be potentially sight threatening due to their propensity to cause infection in view of its direct communication to the anterior chamber. They can be arbitrarily divided into early (less than 3 months) or late (more than 3 months) exposure. Early exposure generally results from improper surgical technique such as inadequate suturing of the implant with implant migration and extrusion, inadequate apposition of scarred or shortened conjunctiva, tight conjunctival closure causing pressure necrosis and conjunctival defect [1]. The causes of late exposure are local ischemia and apoptosis of conjunctiva from micro vascular compression produced by suture materials or immunological responses leading to resorption of plate fixation suture [2], thus causing excessive implant mobility and consequent conjunctival friction. In addition, improper or loose fixation of the plate could also predispose to micro movements, creating constant friction and conjunctival erosion and ultimate extrusion of the plate.

Ayyala et al. [2] retrospectively looked at risk factors for exposure and noted that age, implant location, type of glaucoma, diabetes mellitus, and hypertension had no relation to implant exposure in their series. But the odds of exposure were 9 times higher in eyes with at least one prior intraocular surgery. They hypothesized that previous ocular surgery may induce conjunctival scarring or thinning, and hence fail to preserve the device. Ocular surgery such as vitrectomy or keratoplasty may influence the ocular rigidity making the device more mobile. Use of antifibrotic agents like Mitomycin-C for the prior filtering surgeries would also affect the conjunctival health adversely and would predispose these eyes to conjunctival ischemia and necrosis. Other risk factors that were significantly associated with exposure in a case series by Stephen et al. [4], were diabetes mellitus, black race, number of pre-shunt glaucoma medications, previous glaucoma laser surgery and combined initial implant and cataract surgery. In diabetes, the decrease in microvasculature leads to ischemic conjunctiva that is slower to heal after surgery, less likely to resist the factors causing extrusion and more likely to re-extrude after repair. The exposure rates were also significantly higher in cases with rigid implants and using Polyethylene terephthalate [4] (Mersiline, Ethicon) sutures (5-0 or 6-0), which caused bulkier knots than 10-0 prolene in fixing the plate to sclera. Our series had only AGV silicone implants and none had mersiline sutures, only 10-0 prolene or nylon sutures were used for implant fixation. 

The possible causes of extrusion in our 1^st^ case could be higher number of pre-shunt surgeries, absence of anchoring suture to the plate and the tube and absence of scleral patch graft. In the 2^nd^ case, likely causes were poor anchorage of the implant to sclera and use of a larger sized (FP7) implant in a child resulting in tense and improper conjunctival closure, conjunctival dehiscence and implant extrusion. We acknowledge the fact that these previous surgical inadequacies are difficult to prove, but they were evident on the operating table during revision surgery. Eye rubbing in this child with vernal keratoconjunctivitis also could have predisposed to this problem. In the 3^rd^ and 4^th^ case, it is possible that thin sclera would have precluded proper anchoring of the implant. It is our speculation that possible inflammatory component in these eyes may have caused scleral necrosis and disruption of the anchoring suture leading to exposure though all the systemic investigations that were carried out were within normal limits. We would strongly recommend against using drainage implants in the cases where the sclera itself is involved in the disease process. Extrusion in the 5^th^ case was likely induced following trauma and conjunctival dehiscence. It is probable that the patient was more prone for trauma as he had initially presented with traumatic infectious keratitis. Hence proper follow-up and educating the patient about preventing eye rubbing or injury to the eye are very important in eyes with implants. Conjunctival ischemia from uncontrolled diabetes along with improper anchoring of the implant with conjunctival retraction could be the most likely causes for recurrent plate exposure in the 6^th^ case despite using corneal patch graft and repairing implant extrusion with buccal mucosal graft. In this regard, control of diabetes, adequate anchoring of the implant, liberal conjunctival dissection and closure with minimal tension would have avoided these recurrent exposures.

Management of our individual cases was based on the severity of presentation and the details that are given in Table 1 (Tab. 1). There are several therapeutic options described in literature to manage the exposed implants, such as careful observation without any intervention in case of controlled intraocular pressure and absent infection [1], repositioning the implant at other locations and patching with human scleral graft and amniotic membrane [1], oral buccal mucous membrane in combination with a lamellar corneal patch graft [5] and complete removal with or without reimplantation at a different location [6] or using a preserved pericardial (Tutoplast^®^) [7] plug to repair a corneoscleral fistula after AGV explantation. 

Once a major part of the implant body is exposed, it may be advisable to remove the implant rather than attempting to close it, as chances for necrosis and re-exposure remain very high. In the retrospective study by Yong SooByun et al. [1], four out of seven eyes with implant exposure which required revision surgery with scleral patch graft and amniotic membrane, suffered re-exposure and had to be explanted. Removal and simultaneous replacement of Ahmed glaucoma valves in different quadrants in the same sitting have been described by Michael Smith et al. [6] in a case series of six patients. Four out of the six eyes maintained good intraocular pressure control with medications. One eye needed implantation of a 2^nd^ implant and one eye suffered from hypotony. The authors suggested inserting the 2^nd^ implant before removal of the existing tube to avoid the risk of the eye becoming soft after tube removal.

The reported complications of revision surgery in the literature are phthisis bulbi, recurrent tube erosion, tube migration toward the corneal endothelium and cystoid macular edema [1]. One of the cases in our series treated for extruded implant with removal and reimplantation had a tube erosion 9 months after initial repair, which was treated with scleral patch graft and conjunctival advancement. However, there were no implant plate exposures or extrusion in any of the cases. Long-term meticulous follow-up of these eyes with implant devices are advocated with special care taken to examine the implant and tube area for thinning or erosion and early detection of these complications would prevent sight threatening sequel.

In conclusion exposure of Ahmed glaucoma valve is a serious complication. The possible factors associated with implant exposure in our series were higher number of pre-shunt surgeries, inadequate surgical technique such as improper or absent anchoring suture to the implant plate, absent scleral/corneal patch graft, absent anchoring suture over the tube, improper conjunctival closure, trauma and lastly poorly controlled diabetes. Several management options are also discussed to effectively manage this complication. The cases in our series had variable follow-up. We acknowledge the importance of long follow-up of these eyes as the chance of repeat exposure or loss of intraocular pressure control can be identified with longer follow-up. 

To summarize, in this article we have described our experience in managing extruded implants in six eyes of five patients. The possible causative factors, various options to manage this complication and preventive strategies have been described. 

## Notes

### Competing interests

The authors declare that they have no competing interests.

## Figures and Tables

**Table 1:**
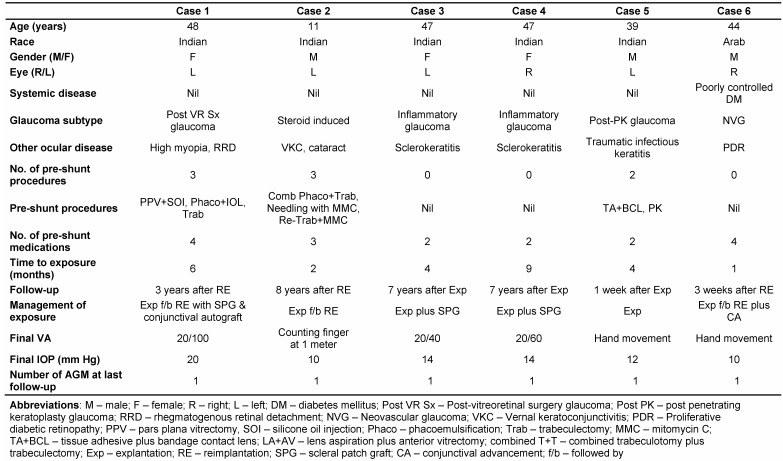


**Figure 1:**
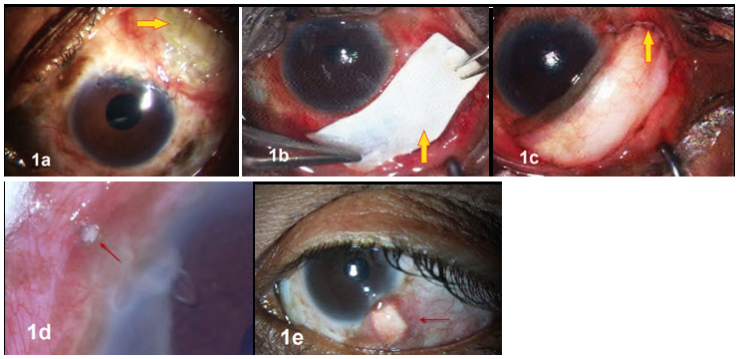
1^st^ case a) at presentation, b) and c) interoperatively, d) 9 months postoperatively, e) 3 years postoperatively

**Figure 2:**
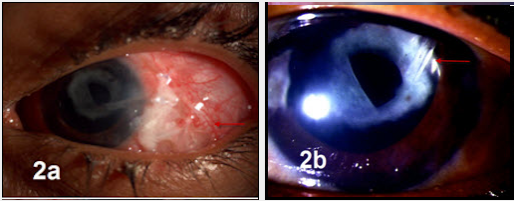
2^nd^ case a) at presentation, b) postoperative

**Figure 3:**
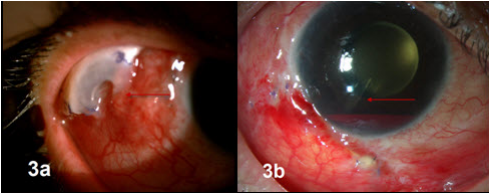
6^th^ case a) at presentation, b) postoperative
